# Linking Chronic Infection and Autoimmune Diseases: *Mycobacterium avium* Subspecies *paratuberculosis*, *SLC11A1* Polymorphisms and Type-1 Diabetes Mellitus

**DOI:** 10.1371/journal.pone.0007109

**Published:** 2009-09-21

**Authors:** Daniela Paccagnini, Lee Sieswerda, Valentina Rosu, Speranza Masala, Adolfo Pacifico, Maria Gazouli, John Ikonomopoulos, Niyaz Ahmed, Stefania Zanetti, Leonardo A. Sechi

**Affiliations:** 1 Dipartimento di Scienze Biomediche, Sezione di Microbiologia clinica e sperimentale, Sassari, Italy; 2 Bay District Health Unit 999, Thunder Bay, Ontario, Canada; 3 Servizio di Diabetologia, Clinica Medica Universitaria di Sassari, Sassari, Italy; 4 Department of Biology, School of Medicine, University of Athens, Athens, Greece; 5 Department of Anatomy-Physiology, Faculty of Animal Science, Agricultural University of Athens, Athens, Greece; 6 Pathogen Biology Laboratory, Department of Biotechnology, University of Hyderabad, Hyderabad, India; Charité-Universitätsmedizin Berlin, Germany

## Abstract

**Background:**

The etiology of type 1 diabetes mellitus (T1DM) is still unknown; numerous studies are performed to unravel the environmental factors involved in triggering the disease. *SLC11A1* is a membrane transporter that is expressed in late endosomes of antigen presenting cells involved in the immunopathogenic events leading to T1DM. *Mycobacterium avium* subsp. *paratuberculosis* (MAP) has been reported to be a possible trigger in the development of T1DM.

**Methodology/Principal Findings:**

Fifty nine T1DM patients and 79 healthy controls were genotyped for 9 polymorphisms of *SLC11A1* gene, and screened for the presence of MAP by PCR. Differences in genotype frequency were evaluated for both T1DM patients and controls. We found a polymorphism in the *SLC11A1* gene (274C/T) associated to type 1 diabetic patients and not to controls. The presence of MAP DNA was also significantly associated with T1DM patients and not with controls.

**Conclusions/Significance:**

The 274C/T *SCL11A1* polymorphism was found to be associated with T1DM as well as the presence of MAP DNA in blood. Since MAP persists within macrophages and it is also processed by dendritic cells, further studies are necessary to evaluate if mutant forms of *SLC11A1* alter the processing or presentation of MAP antigens triggering thereby an autoimmune response in T1DM patients.

## Introduction

Type 1 diabetes mellitus (T1DM) is a multifactorial autoimmune disease in which T-lymphocytes infiltrate the islets of the pancreas and destroy the insulin-producing beta cell populations [1]. The exact cause of T1DM is not clearly known. However T1DM constitutes interactions of polygenic traits with environmental factors that are not clearly defined in the available literature and it is not known what triggers autoimmunity to self-antigens such as those expressed in the pancreatic islets of Langerhans cells [2], [3].

Accumulating line of evidence points to role for *Mycobacterium avium* subsp. *paratuberculosis* (MAP) in the development of T1DM as an environmental trigger [2], [4], [5]. MAP bacteria have been generally known to harness molecular mimicry as a strategy to avoid clearance [6]. Recently our group observed immune responses to MAP in T1DM patients, thus supporting an infectious cause for T1DM [7]. Moreover, the presence of MAP was confirmed in T1DM patients by culture and was isolated from blood of T1DM patients [4]. It has long been held that genetic susceptibilities, epitope homologies, and endemic bacterial load in the environment might support the case for an infectious trigger, such as MAP, to be the probable agent of T1DM in genetically susceptibility individuals [3], [8], [9], [10].

Regarding genetic susceptibility, the *SLC11A1* gene (previously known as *NRAMP1*) is a functional and a positional candidate gene that associates with T1DM as well as susceptibility to mycobacterial infections [11], [12], [13], [14]. Additionally, Kissler *et al*. [15] demonstrated that *SLC11A1* gene silencing using RNAi approach in mice reduced the frequency of T1DM and protected against experimental autoimmune encephalomyelitis, advocating thereby for a role for *SLC11A1* in autoimmunity. Moreover, it was recently demonstrated that association of variants of the gene encoding *SLC11A1* with T1DM may reflect its function in processing and presentation of islet cell self-antigens to dendritic cells (DCs) [16].

Thus, non-MHC genes could affect the MHC-restricted T-cell response through altered antigen processing and presentation.

To date, a number of polymorphisms at the *SLC11A1* locus have been associated with susceptibility to infectious agents and to autoimmune disorders [17]. Specifically, a 5′(GT)n repeat polymorphism in the promoter region of the *SLC11A1* gene seems to be of particular interest, since it has been shown to affect the levels of gene expression [17]. *In vitro* studies of this polymorphism suggested direct contribution of particular alleles either to autoimmune (allele 3) or to infectious (allele 2) disease susceptibility [10], [17]. Nevertheless, variants of the *SLC11A1* located within the coding region, the introns, and the 3′-UTR have been shown to influence susceptibility to autoimmune disorders and T1DM [13], [18].

Our study aimed at examining the association of the *SLC11A1* polymorphisms in relation to the presence of MAP infection, with T1DM in patients from Sardinia.

## Methods

### Patients and controls

A total of 131 participants comprising of 59 T1DM patients (28 females and 31 males with age ranging between 18–94 years) and 76 healthy controls (Table 1) were tested for the detection of *SLC11A1* polymorphisms and the presence of MAP specific IS900 signature using total DNA extracted from peripheral blood mononuclear cells collected at the Institute of Diabetology, medical clinic of Sassari University, Italy. Informed written consents from patients including other necessary clearances were obtained before blood samples were drawn. Institutional review board of the University of Sassari approved the study.

**Table 1:** Presence of MAP and *SLC11A1* genotypic frequencies in patients with type 1 diabetes mellitus and non-diabetic controls.

		Type 1 diabetes mellitus (n = 59)	Non-diabetic (n = 79)	p-value*
MAP**	present	33	18	
	not present	26	61	<0.0005
274 C/T	Allele 1	12	35	
	Allele 2	28	30	
	Allele 1 & 2	19	7	<0.0005
	No product	0	7	
469+14G/C	Allele 1	30	36	
	Allele 2	2	3	
	Allele 1 & 2	3	15	0.065
	No product	24	25	
577-18G/A	Allele 1	0	4	
	Allele 2	59	72	
	Allele 1 & 2	0	0	0.131
	No product	0	3	
823C/T	Allele 1	0	0	
	Allele 2	44	69	
	Allele 1 & 2	0	1	1.000
	No product	15	9	
A318V	Allele 1	0	2	
	Allele 2	40	46	
	Allele 1 & 2	0	0	0.498
	No product	19	31	
1465-85G/A	Allele 1	25	35	
	Allele 2	20	16	
	Allele 1 & 2	13	8	0.196
	No product	1	20	
D543N avaII	Allele 1	1	1	
	Allele 2	57	68	
	Allele 1 & 2	1	0	0.730
	No product	0	10	
D543N fokI	Allele 1	46	59	
	Allele 2	0	1	0.9517
	Allele 1 & 2	0	0	
	No product	13	19	
1729+55del4	Allele 1	59	46	
	Allele 2	0	0	
	Allele 1 2	0	0	1.000
	No product	0	33	
(Gt)n	Allele 3	31	21	0.5983
	Allele 2	9	9	
	Altro 3*/2*	6	8	0.5912
	no product	13	41	

* Fisher's exact test;

** MAP  =  Mycobacterium avium subspecies paratuberculosis.

No product =  no amplification obtained.

Briefly, 5 ml blood from patients was centrifuged and plasma supernatant used in ELISA. Remaining plasma samples were aliquoted and stored frozen at −20°C for short-term storage (<6 months) and −80°C for long term storage (>6 months).

### DNA extraction

To extract MAP DNA, samples were processed by using the lysor instrument - 500 µl of glass beads (Sigma) were added, followed by standard phenol-chloroform extraction and DNA precipitation with absolute ethanol and 10M ammonium acetate; the pellet was washed with 70% ethanol and resuspended in 70 µl of TE buffer.

Human genomic DNA extraction for *SCL11A1* gene amplification was performed as previously published [14].

### MAP DNA detection

MAP DNA detection was performed by PCR as previously reported [14], [19]. Sequence analysis of the amplicons confirmed IS900 identity. For *SCL11A1* polymorphisms, 100 ng of template (genomic) DNA, obtained from blood cells, was amplified. The primer sequences and PCR cycling parameters that we used were previously reported [5].

### *SLC11A1* genotyping

Nine polymorphisms (-274C/T, D543N G/A, 823C/T, -237C/T, INT4G/C, 577-18G/A, A318V C/T, 1465-85G/A, and 1729+55del4) were genotyped across *NRAMP1* [14]. Candidates were genotyped across these polymorphisms by PCR restriction fragment length polymorphism (PCR-RFLP), in which primers and restriction enzymes were used as previously described [14]. Restriction enzyme digestion products were resolved by electrophoresis on 3% agarose gels stained with ethidium bromide.

Statistical analysis was performed by using the Chi square test with Yate's correction. Inference was aided by GraphPad InStat (version 3.00, GraphPad Software, Inc., San Diego, CA, USA)

## Results

### MAP detection

MAP DNA was amplified from the blood of 33 out of 59 T1DM patients (55.9%) whereas MAP was detected only in 18 out of 79 healthy controls (22.7%) indicating a statistically highly significant difference (Table 1).

### *SLC11A1* allele frequencies

*SLC11A1* allele frequencies for 274 C/T polymorphism differed significantly between T1DM patients and control group, this polymorphism was significantly associated with diabetes type 1, in particular allele 1 and 2 generated a P value of <0.0005 (Table 1).

There was no significant difference in the allele frequencies of the other 8 polymorphisms for 823 G/T, 577-18G/A, A318V and 1465-85G between T1DM patients and control group (Table 1).

Bivariate and multivariate logistic regression analysis was very significant both for the presence of MAP and *SLC11A1* 274C/T genotype to predict diabetes status among patients with T1DM and non-diabetic controls as shown in Table 2. In particular, allele 2 alone showed an odds ratio of 2.7 (1.2, 6.3) in a bivariate model and 2.7 (1.2, 6.3) in a multivariate model whereas allele 1 showed an odds ratio of 7.9 (2.7, 23.5) in a bivariate model and 9.4 (2.9, 30.3) in a multivariate model.

**Table 2:** Bivariate and multivariate logistic regression analysis using the presence of MAP and *SLC11A1* 274C/T genotype to predict diabetes status among patients with type 1 diabetes mellitus and non-diabetic controls.

		Bivariate model	Multivariate model
Predictor		OR (95% CI) *	OR (95% CI)
MAP**	Not present	Ref***	Ref
	Present	4.3 (2.1, 9.0)	4.7 (2.1, 10.7)
274C/T	Allele 1	Ref	Ref
	Allele 2	2.7 (1.2, 6.3)	3.0 (1.2, 7.5)
	Allele 1 & 2	7.9 (2.7, 23.5)	9.4 (2.9, 30.3)

* OR (95% CI)  =  Odds ratio (95% confidence interval).

** MAP  =  Mycobacterium avium subspecies paratuberculosis.

*** Ref  =  reference category.

## Discussion

*SLC11A1* gene has a vital function in the pathway of macrophage activation [20]. It is involved in the expression of chemokines, interleukin-1β, inducible nitric oxide synthase, MCH II molecules, and TNF-α [17], [21]. The localization of SLC11A1 is within the late endosomal/lysosomal compartments in phagocytes and it has been hypothesised to perform a transporter function as a divalent cation [20], [22]. Recent reports show a role of the *SLC11A1* gene in different autoimmune diseases such as Crohn's disease [23], rheumatoid arthritis and juvenile rheumatoid arthritis [24], [25] multiple sclerosis [26], diabetes type 1 [18] and infectious diseases including tuberculosis [27], [28], [29] and leprosy [30], [31]. There is evidence that *SLC11A1* gene is involved in modifying susceptibility to T1DM as shown by RNA silencing that inversely correlated with increased susceptibility to infection [13].

Antigen presenting cells have long been considered associated to diabetes susceptibility [32], [33] due to their exclusive expression of MHC class II molecules, the major genetic factors underlying T1DM. Different workers have identified diabetes-associated genes that are also involved in antigen-processing pathways [8], [34]. SLC11A1 functions as a membrane transporter of divalent cations but its mechanism of mediating natural resistance to bacterial and parasitic infections remains unclear [17], [22]. Previous studies on the innate response of macrophages during intracellular bacterial infection suggested that SLC11A1-mediated deprivation of divalent cations might change the phagosomal microenvironment that impairs the pathogenesis of intracellular pathogens [20]. Dai *et al.* found that SLC11A1 contributes to enhanced phagosomal acidification in macrophages and dendritic cells [16]. Based on these data, Dai *et al.* [16] suggest that SLC11A1-mediated changes in antigen processing within the vesicular system may have general effects in autoimmune as well as in infectious diseases.

MAP is the causative agent of Johne's disease in ruminants [35] and it has been associated with Crohn's disease [35], [36]. We have previously reported the presence of MAP DNA in the PBMC of T1DM patients [5]. These finding were confirmed by detecting MAP specific antibodies in the blood of T1DM patients but not in type 2 diabetic patients and controls [19]. More recently two MAP strains were isolated from the blood of T1DM patients by our group [4].

In this study we report the novel association of T1DM with the 274 C/T polymorphism within the *SLC11A1* gene and the presence of MAP DNA in Sardinian T1DM patients. This polymorphism may be the link of a permissive infection by MAP in these people. Recent studies have reported that the 4-bp TGTG deletion located 55 nucleotides downstream of the last codon in exon 15 (1729 +del55del4) is associated with tuberculosis susceptibility in Chinese children [29]. We did not find any statistical difference at this locus between T1DM and healthy controls. *SCLA11* polymorphisms were analyzed also by stratification by sex and no differences were observed (data not shown). It may be possible that polymorphisms in different loci of the *SCL11A1* gene may confer susceptibility to different intracellular pathogens although genetically very close. Further studies are necessary in order to elucidate the role of *SCL11A1* polymorphisms and how it may influence MAP infection in humans.
